# A Distributed Particle-Swarm-Optimization-Based Fuzzy Clustering Protocol for Wireless Sensor Networks

**DOI:** 10.3390/s23156699

**Published:** 2023-07-26

**Authors:** Chuhang Wang

**Affiliations:** College of Computer Science and Technology, Changchun Normal University, Changchun 130032, China; wangchuhang@ccsfu.edu.cn

**Keywords:** clustering, fuzzy logic, particle swarm optimization, energy efficiency, network lifetime

## Abstract

Clustering is considered to be one of the most effective ways for energy preservation and lifetime maximization in wireless sensor networks (WSNs) because the sensor nodes are equipped with limited energy. Thus, energy efficiency and energy balance have always been the main challenges faced by clustering approaches. To overcome these, a distributed particle swarm optimization-based fuzzy clustering protocol called DPFCP is proposed in this paper to reduce and balance energy consumption, to thereby extend the network lifetime as long as possible. To this end, in DPFCP cluster heads (CHs) are nominated by a Mamdani fuzzy logic system with descriptors’ residual energy, node degree, distance to the base station (BS), and distance to the centroid. Moreover, a particle swarm optimization (PSO) algorithm is applied to optimize the fuzzy rules, instead of conventional manual design. Thus, the best nodes are ensured to be selected as CHs for energy reduction. Once the CHs are selected, distance to the CH, residual energy, and deviation in the CH’s number of members are considered for the non-CH joining cluster in order to form energy-balanced clusters. Finally, an on-demand mechanism, instead of periodic re-clustering, is utilized to maintain clusters locally and globally based on local information, so as to further reduce computation and message overheads, thereby saving energy consumption. Compared with the existing relevant protocols, the performance of DPFCP was verified by extensive simulation experiments. The results show that, on average, DPFCP improves energy consumption by 38.20%, 15.85%, 21.15%, and 13.06% compared to LEACH, LEACH-SF, FLS-PSO, and KM-PSO, and increases network lifetime by 46.19%, 20.69%, 20.44%, and 10.99% compared to LEACH, LEACH-SF, FLS-PSO, and KM-PSO, respectively. Moreover, the standard deviation of the residual network was reduced by 61.88%, 55.36%, 54.02%, and 19.39% compared to LEACH, LEACH-SF, FLS-PSO, and KM-PSO. It is thus clear that the proposed DPFCP protocol efficiently balances energy consumption to improve the overall network performance and maximize the network lifetime.

## 1. Introduction

Wireless sensor networks (WSNs) consist of numerous tiny, low-cost sensor nodes that are randomly deployed in the predetermined target area. Although WSNs have been widely used in applications such as target tracking, disaster prevention, and space exploration [1], recharging or replacing the nodes are usually costly or even impossible in harsh application environments. Thus, energy saving for nodes powered by batteries has always been extremely important for WSNs to prolong their network lifespan as much as impossible [1,2]. Clustering has been extensively validated to be an effective method for reducing energy, increasing scalability, decreasing delay, and enhancing network lifetime [3]. 

**Figure 1:**
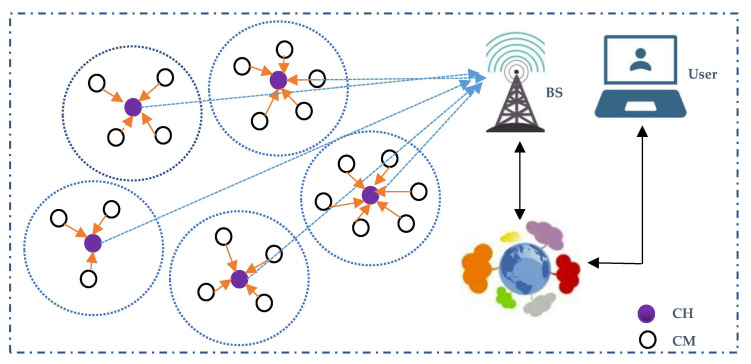
Illustration of a clustered WSN.

Clustering is used to dynamically organize the network by dividing the nodes into a certain number of clusters [3,4]. In a cluster, a node with the best resources is selected as the cluster head (CH), which is responsible for cluster management, data collection, aggregation, and transferring, while the other nodes become cluster members (CMs) and send their sensed data to the CHs. Moreover, the base station (BS) receives the transferred data from all the CHs for further processes. Accordingly, the cluster-based network is shown in Figure 1. As shown in the figure, the CHs play a critical role in the network as a bridge between CMs and the BS. Therefore, CH selection is a critical issue in clustering protocols, and is considered to be an NP-hard problem [5]. In the state-of-the-art clustering protocols, computational intelligence algorithms based on the genetic algorithm [6], gray wolf optimization [7], particle swarm optimization [8], bacteria foraging optimization [9], and fuzzy logic [10] are increasingly being used to find the optimal solution. In particular, compared with non-fuzzy clustering approaches [11,12], fuzzy-logic-based schemes can better handle the uncertainties inherent in clustering [13], and provide more flexibility and a better combination of input parameters, so as to achieve the optimal solution [14]. Once the CHs are selected properly, the other non-CH nodes find their specific CHs based on factors such as distance and residual energy to finally form clusters through message interaction between them [4,12]. Although non-CH nodes’ joining clusters are sometimes integrated with CH selection using a genetic algorithm [15], fuzzy c-means [14], and AP [16] to save energy, their centralized clustering mechanism not only reduces the scalability for larger network applications, but also increases the computational complexity and number of control messages. Finally, a TDMA schedule, such as that in the pioneer clustering protocol LEACH [4], is usually utilized to further reduce the intra-cluster energy consumption, and data transmission in single-hop [4,13,14] or multi-hop [10,15,16] modes is employed to collect data in the network.

Generally, a fuzzy logic system consists of a fuzzifier, inference engine, knowledge base, and defuzzifier, which can make the best possible decision in scenarios characterized by uncertainty [10,16]. Due to its low complexity and high suitability, fuzzy logic systems with different descriptors have been widely used in the WSN domain, including clustering [10], routing [16], clustering and routing [17], attack detection [18], data aggregation [19], and trust node detection [20]. For clustering, a fuzzy logic system is usually utilized to select the CH, calculate the competition radius, nominate the CH for CMs, and determine re-clustering [21]. In order to make better decisions, the parameters in the fuzzy logic system are usually well-integrated by assigning appropriate membership functions and determining the result by setting different fuzzy rules. However, it is almost impossible to make the best decision using manual rule generation in traditional protocols based on expertise and practice, and this is especially unsuitable for different applications. Thus, several optimization algorithms, such as the genetic algorithm [22], shuffled frog algorithm [23], particle swarm optimization [13], and artificial bee colony [14], have been adopted to tune the rule base. After the CH is selected and the CMs have joined the clusters, the clusters are formed, followed by the data transmission process, which is usually completed by a routing algorithm or protocol [17]. Conventionally, a periodic re-clustering mechanism based on the round, which is defined by the time from the beginning of clustering to the end of all source nodes sending data to the BS, is used to maintain the clusters after the data transmission process [4,21]. Although a few methods based on adaptive round length [24] have verified its better effectiveness compared to those with a fixed round length, by significantly decreasing the number of rotating CHs, it is still hard to obtain the best round length due to the influence of network dynamics and uncertainties [16]. Therefore, an on-demand re-clustering mechanism using a fixed round length has been adopted to conduct non-periodic clustering, which can significantly save energy consumption to extend the network lifetime [25,26]. However, there are still some key problems with the existing solutions, including the neglect of suitable integration of energy efficiency and balancing, fixed fuzzy rules used only for a specific application, and global decisions on re-clustering. Therefore, significant room is left for improvement.

In this paper, a distributed particle swarm optimization-based fuzzy clustering protocol called DPFCP is presented to select the best CHs, form optimal clusters, and maintain the clusters by addressing all the problems and assimilating all the benefits emphasized above. In DPFCP, energy-efficient and balanced clusters are formed through fuzzy CH selection and non-CH joining clusters based on the respective, carefully considered parameters. Moreover, the fuzzy rules can be adaptively tuned according to different applications based on a particle swarm optimization algorithm. Another advantage of DPFCP is its on-demand re-clustering mechanism, which maintains clusters not only globally, but also locally, so as to further reduce energy dissipation. Accordingly, the network lifetime is maximized in DPFCP. Contributions of the proposed protocol are as follows:

- A clustering protocol to form optimal clusters and select the best CHs by utilizing a PSO-based fuzzy logic system considering effective parameters.
- Fuzzy rules generated by particle swarm optimization, and non-CH nodes joining clusters considering not only distance, but also residual energy and node degree.
- An on-demand re-clustering mechanism to maintain clusters locally and globally to reduce computation and message overheads.

The remainder of this paper is organized as follows. Related works on fuzzy clustering protocols are described in Section 2, and the system model is addressed in Section 3. The detailed design of the proposed protocol is provided in Section 4. Section 5 presents the simulation results. Finally, conclusions and future directions are given in Section 6.

## 2. Related Works

LEACH was the first clustering protocol proposed to balance the energy consumption by organizing nodes into clusters based on probability [4]. Although numerous approaches have been presented to improve the performance of LEACH, its main idea has been inherited and is widely used today [1]. Fuzzy logic has been proven to be applicable to almost all aspects of clustering from CH selection, cluster radius calculation, CM joining cluster, next-hop CH finding, re-clustering, etc. [16,21,27]. However, routing protocols are usually responsible for finding suitable next-hop CHs or paths for data transmission in multi-hop mode [16,23]. In addition, clustering protocols dedicated to forming clusters with single-hop data transmission can be easily integrated with routing methods as clustering and routing protocols [1,17]. Hence, only single-hop fuzzy clustering protocols are the focus here.

In [28], LEACH-FL uses a Mamdani fuzzy system to select CHs. Every round, the presented fuzzy logic system in each node takes the battery level of the node, the node density, and the distance from the BS into consideration. This means that the higher the battery level of the node, the denser the node density, and the smaller the distance from the BS, the greater the probability the node becomes a CH. After fuzzy inference based on IF–THEN rules and the inputs, the final crisp output probability value can be obtained by defuzzification of the center of area (COA). In contrast to LEACH selecting CH in a probabilistic manner by comparing a randomly given number with a threshold value, LEACH-FL compares the probability value with the threshold value defined in LEACH, and if it is less than the threshold value, the node becomes a CH; otherwise, it becomes a CM. The next steps are the same as those of LEACH: clusters are formed by message interaction between CMs and CH, and data transmission is achieved by using TDMA. Simulation results indicate that LEACH-FL outperforms LEACH in terms of network energy consumption and lifetime.

In [29], FUCA is used to solve the hot spot problem by using a Mamdani fuzzy logic system. At first, the preliminary CHs are selected among all the nodes by a probability-based method in LEACH, i.e., performing a comparison between a random number with the threshold. Then, a fuzzy logic system is employed to calculate the competitive radius and rank for the preliminary CH. Three parameters, namely, the distance to the BS, residual energy, and the density of nodes, are used for inputs of the fuzzy logic system. The fuzzy inference engine processes the linguistic variables of the three input parameter according to the IF–THEN rules. Moreover, a defuzzifier based on COA maps the output linguistic variable into crisp competitive radius and rank values. Finally, nodes with higher ranks are selected as final CHs, which also should satisfy only one CH within its competitive radius. Next, each CH broadcasts an advertisement message announcing its CH identity, and non-CH nodes join the closest CH by the feedback of a joining message after they receive a confirmation message from CH nodes; thus, the clusters are formed. Finally, CMs send sensed data to their corresponding CHs, and CHs forward the fused data to the sink. Simulations results show that FUCA can form unequal clusters to balance the network energy consumption and extend the network lifetime.

In [30], LEACH-FC is proposed to enhance the network energy efficiency and balance the energy load at each node. First, a Mamdani fuzzy logic system with descriptors of node energy, node concentration, and node centrality is used to calculate the CH chance value, and the larger the chance value of a node, the more likely it is to become a CH. Of course, it also has higher energy, higher concentration and closer centrality. Next, another Mamdani fuzzy logic system is utilized to find an appropriate CH for each non-CH node, which evaluates the factors of node energy, distance to BS, and distance to the CH to make a decision about which CH it belongs to. Moreover, a vice-CH in each cluster is selected to improve the reliability of the cluster according to its second maximum chance value. Finally, the BS broadcasts a message including the CH’s ID, the vice-CH’s ID, and the CM’s ID in each cluster. Then, clusters are formed while all the nodes receive this message. Simulation results show that LEACH-FL largely improves the network lifetime and reliability in both homogeneous and heterogeneous WSNs.

In [31], FEECS is presented to prolong the network lifetime using fuzzy-logic based-CH selection. K-means is used to form a predetermined number of clusters in an efficient way. Afterwards, a Mamdani fuzzy system is adopted to select optimal CHs based on the parameters residual energy, node degree, distance to the BS, and cluster status, indicating whether the CH candidate plays a CH role. These four inputs and IF–THEN rules are used by the fuzzy inference engine to infer the output fuzzy set, and the fuzzy set is mapped into a crisp output chance through COA defuzzification, indicating the goodness of being a CH. Finally, the node with the maximum chance value becomes a CH, and announces its identity to its CMs. Moreover, TDMA is used to avoid collisions among intra-cluster data transmission. Simulation results show that FEECS can efficiently enhance the network energy utilization and extend the longevity of the network’s lifetime.

In [10], a fuzzy clustering method is proposed to improve the network energy efficiency and boost the network throughput, which is called KM-PSO for simplicity. Firstly, a preset number of clusters are formed by K-means using Euclidean distance as a similarity. Moreover, particle swarm optimization is used to find the optimal cluster centers based on a fitness function considering maximization of distance between nodes to the cluster centers. Secondly, the primary CH (PCH) and secondary CH (SCH) in each cluster are determined by two Mamdani fuzzy logic systems whose inputs are the node’s residual energy, distance from the node to the cluster’s center, distance from the node to the BS, the node’s energy level, and the distance from the node to the PCH within its cluster, respectively. Through fuzzy inference based on IF–THEN rules and defuzzification of COA, like in LEACH, LEACH-FC, and LEACH-SF, the crisp probability of being a PCH and SCH is obtained. Moreover, the two nodes with the maximum probability become the PCH and SCH in each cluster, respectively. After cluster formation with the selection of the PCH and SCH, the BS broadcasts a message including the IDs of the PCH, SCH, and related CMs. For data transmission, CMs send their data to the SCH based on TDMA, and the SCH receives and aggregates the data and transfers it to the PCH. After that, the PCH forwards the data to the BS. Moreover, in contrast to the other protocols, KM-PSO runs hybrid K-means and PSO cluster formation only once, while fuzzy selection of the PCH and SCH, and intra-cluster data communication, are conducted in each round. Simulation results prove that the network lifetime and packet transmission are both significantly improved.

In [32], a fuzzy clustering scheme is presented to enhance the network lifetime. Fuzzy c-means is used to determine the clusters’ centers and their associated member nodes at the sink. In the first round, the sink nominates CHs from the centers and sends an advertisement message to the network. Then, the CHs also broadcast message to announce their CH identity, and the other nodes join the nearest CH after hearing the message from the CHs to form clusters. Moreover, each node calculates its fitness value using a Mamdani fuzzy inference system with the descriptors of residual energy and distance to the cluster center. The inference engine of the fuzzy logic system converts the two fuzzified input variables into fuzzy output based on the *IF–THEN* rules. The aggregated fuzzy set output is then defuzzified using the COA method to obtain the crisp fitness value. The fitness value of each node is also sent to the sink by the corresponding CH at the end of each round. Afterwards, the sink selects the node with the highest fitness as the CH among a cluster center associated with the member nodes. Similarly, all the best CHs can be selected. Then, the sink broadcasts an advertisement message including the CHs selected by fuzzy logic instead of random nomination. This process continues in the following rounds until all the nodes die. Simulation results show that the proposed scheme can reduce the number of control packets and improve the network lifetime.

In [14], LEACH-SF is presented to achieve energy efficiency in different practical heterogeneous wireless sensor networks. Balanced clusters in LEACH-SF are formed using fuzzy c-means with the objective function minimizing the distance between nodes and the cluster centroid. Once the clusters are formed, a Sugeno fuzzy logic system is used to calculate an impact factor (IF) within [0, 1] for each node based on residual energy, distance from the sink, and distance from the cluster centroid. Moreover, an artificial bee colony algorithm is used to adjust the fuzzy rules, whose fitness function considers the lifetime in different applications. Consequently, the optimal IF is obtained for each node, and the larger the IF, the higher the priority to be selected as a CH. Finally, the node with the maximum IF becomes the CH of each cluster. The operation and calculations mentioned before are completed by the sink. After CH selection, the sink broadcasts an advertisement message to the CHs with their CH identity and included members, and CHs inform their CMs about their IDs and their allotted timeslot. Simulation results validate that LEACH-SF can not only efficiently form balanced clusters but also maximize the network lifetime.

In [25], an on-demand fuzzy clustering algorithm is presented to improve the network energy efficiency and throughput. At first, a new threshold function is defined to select probable candidate CHs considering more parameters than LEACH, including nodes’ residual energy and optimal number of clusters, which ensures that the responsibility of being a CH is rotated among all the nodes and the nodes with higher residual energy are elected to be CHs. In order to improve the network performance, a Mamdani fuzzy logic system is used to calculate chance for each node, which uses node degree, node centrality, and packet drop probability as descriptors. Moreover, PSO with a fitness function maximizing the chance value is adopted to obtain the best ranges of membership functions for inputs and the output of the fuzzy logic system. Finally, a candidate CH with a higher chance value is selected as the final CH. Once the final CHs are selected, the other nodes become cluster members and join the nearest CH based on the received signal strength. For data transmission, the CMs transmit sensed data to their respective CH based on the TDMA scheme, and the CHs receive and aggregate the data and forward it to the BS. For convenience, the proposed approach is named FLS-PSO. Finally, only when the CH’s residual energy is lower than a threshold defined by $\gamma E_{initial}(0<\gamma <1)\;(E_{initial}$ is the initial energy of the nodes), the CH sends a message to the BS, who is responsible for informing all the nodes to perform re-clustering. Simulation results show that the proposed algorithm can reduce the network energy consumption and improve the packet delivery ratio. A summary of the above-mentioned is presented in Table 1.

## 3. System Model

### 3.1. Network Model

A large number of nodes are randomly scattered in the clustered network, as shown in Figure 1. All the homogeneous nodes remain stationary once deployed, and can directly communicate with the BS. The BS can be located everywhere inside or outside of the network area, which has enough information about the nodes. Moreover, a round is used to periodically collect nodes’ data. In each round, CMs send their sensed data to the corresponding CHs, and CHs receive and aggregate the data and transfer it to the BS. Moreover, some specific hypotheses are made regarding the network:

- All the nodes can be identified by their ID number;
- The BS is also static and has no constraints on energy and other resources;
- Wireless links between nodes (including the BS) are symmetric;
- All the nodes are aware of their position through the received signal strength indicator (RSSI);
- Each node has the capability to adjust its transmission power.

### 3.2. Energy Model

The same first-order radio communication as used in [4,16] is utilized to calculate the dissipated energy in this paper. Based on the transmission distance, the model is separated into two channels, i.e., the free-space and multi-path models. When the transmission distance is greater than a predetermined threshold value $d_{0},$ the multi-path model is used; on the contrary, the free space model is adopted. Consequently, when a node *i* sends l-bit data to a node *j* with the transmission distance *d*, the energy consumption can be calculated by:

(1) $$E_{Tij}(l,d)=\left\{\begin{array}{c} l*E_{elec}+l*\varepsilon_{fs}*d^{2},\;d<d_{0}\\ l*E_{elec}+l*\varepsilon_{fs}*d^{4},d\geq d_{0} \end{array}\right.$$

where $E_{elec}$ is the energy required to transmit or receive 1-bit data, $\varepsilon_{fs}$ and $\varepsilon_{mp}$ denotes the amplifier coefficients of free space and multi-path fading, respectively, and $d_{0}$ is the threshold transmission distance given by $d_{0}=\sqrt{\varepsilon_{fs}/\varepsilon_{mp}\;}$. Moreover, the energy consumption for node *i* to receive *k*-bit data from node *j* can be calculated according to Equation (2):

(2) $$E_{Rij}=k*E_{elec}$$

In addition, the energy consumption for aggregating l-bit data is shown as:

(3) $$E_{DA}=k*E_{pDb}$$

where $E_{pDb}$ is the energy consumption needed to fuse one bit.

## 4. Proposed Protocol

In this section, the proposed DPFCP is presented to handle the clustering problem, which is labeled as one of the most challenging tasks in WSN. This can be divided into three phases: CH selection, cluster formation, and on demand re-clustering. A round is still used to operate DPFCP, and the first two phases are only performed in a few rounds, while the last phase is performed in each round. The details of each phase of DPFCP are elaborated as follows.

### 4.1. CH Selection

Fuzzy logic has shown its superiority in decision making for applications with an uncertain and chaotic environment, and can be used to more effectively deal with the uncertainties in CH selection for derivation of better solution. Usually, the fuzzy logic system uses fuzzy rules to evaluate fuzzy inputs and reason to obtain the fuzzy output. The fuzzy rule base is the set of rules designed to calculate chance or probability of a node to become a CH. The most popular fuzzy inference mode is Mamdani, which uses different inputs and rules to achieve desired results.

In the proposed DPFCP, a fuzzy logic system with the Mamdani model is utilized to select the appropriate candidate CH based on the optimized fuzzy rules. Residual energy, node degree, distance to the BS, and distance to the centroid are considered as fuzzy inputs to avoid the nodes’ premature death (the first input), to minimize the intra-cluster energy consumption (the second input), to balance the intra-cluster energy consumption (the third input), and to reduce the total energy consumption of CHs (the fourth input).

- Residual energy: indicates the remaining energy of node *i* denoted by $E_{res}$ in the current round. It is the first and most important factor considered because of the greater energy consumption needed for a CH to make the cluster operate properly. So, the higher the residual energy, the greater the chance for a node to be selected as a CH and the longer the network lifetime.
- Distance to centroid: indicates the Euclidean distance between node *i* and the centroid of *i* and its neighbors expressed by $E_{to}C$. Distance to the centroid denotes how centrally the node *i* is located in the cluster. The smaller its value, the less energy consumption in the cluster, and the higher the node’s chance of being selected as a CH.
- Node degree: indicates the number of neighbors in the communication range of node *i* denoted by $N_{dn}$. The closer the node degree to the average node degree of the network, the more uniform the cluster and the more likely the node to become the cluster head, which can also help to avoid isolated nodes and a node’s premature death due to the large intra-cluster overhead.
- Distance to the BS: indicates the distance between node *i* and the BS expressed by $D_{to}BS$. The nodes farther from the BS need more energy to forward data than the closer ones. Thus, introducing distance to the BS as one of the decision factors is an effective solution to decrease the network energy consumption. The shorter the distance, the higher the chance of being selected as a CH.

**Figure 2:**
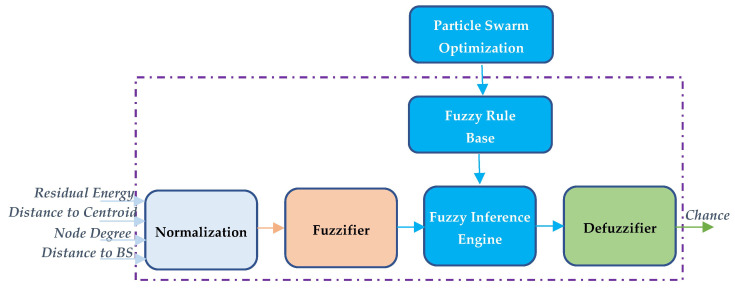
Fuzzy logic system for DPFCP.

Using the above-mentioned inputs, the FLS outputs chance to indicate the priority of a node becoming a CH, which includes normalization, fuzzifier, fuzzy inference engine, fuzzy rule base, and defuzzifier, as shown in Figure 2.

As shown in Figure 2, the discrete values for inputs $E_{res},$ $E_{to}C,$ $N_{dn},$ $D_{to}BS$ are taken in their normalized form so as to put them in the same range. The fuzzifier is then utilized to transform the normalized values into fuzzy input sets using membership functions. The fuzzy inference engine uses *IF–THEN* rules in the fuzzy rule base to map fuzzy input sets for fuzzy output sets. Finally, the defuzzifier is employed to transfer fuzzy output sets to crisp values. Moreover, the fuzzy rule base table is tuned by PSO for optimal network performance based on application. The detailed description of the FLS for DPFCP is presented in the following.

Normalization is used to put similar data from different domains in the same domain, so as to prevent the significant effect of very large input data on the output. Then, the normalized input variables range within [0, 1], which can be expressed as:

(4) $$v_{i}=\frac{v_{i}-v_{min}}{v_{max}-v_{min}}$$

where $v_{i}$ is the crisp value of the input variable v for node *i*, $v_{max}$ and $v_{min}$ are the maximum and minimum value of v in the cluster in which node *i* is located.

**Figure 3:**
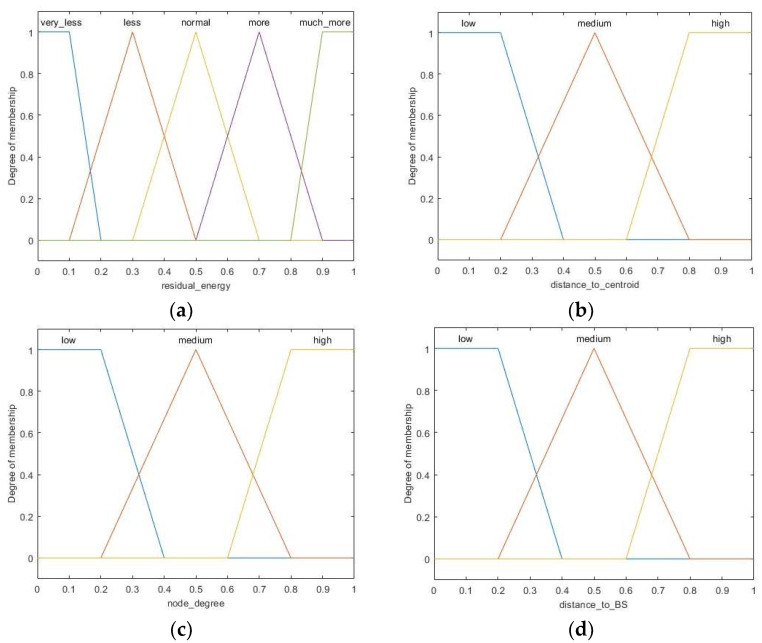
Membership functions for input variables: (**a**) residual energy, (**b**) distance to centroid, (**c**) node degree, (**d**) distance to BS.

**Figure 4:**
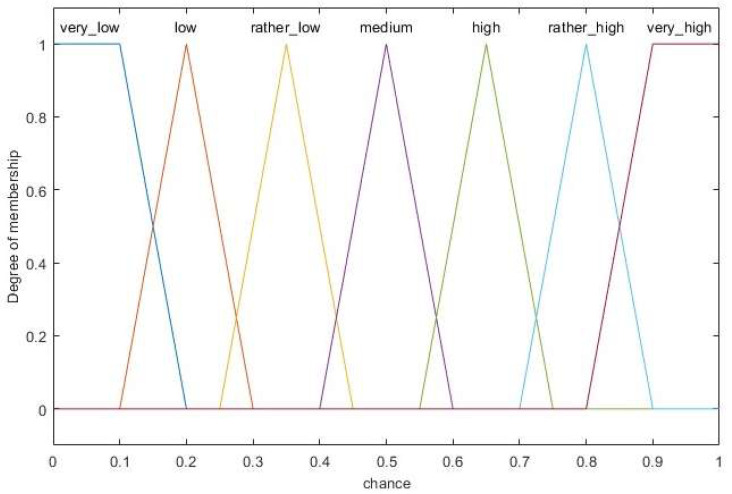
Membership function for output variable.

The fuzzifier maps the crisp normalized inputs to linguistic fuzzy variables using different membership functions. Generally, trapezoidal and triangular membership functions are widely used for fuzzy logic systems because of their faster calculation and simpler implementation. Moreover, the former is usually used for boundary variables while the latter is used for intermediate variables [33]. Due to the importance of input residual energy and the greater flexibility of fuzzy rules, five membership functions are used for this variable, which are described by five linguistic variables: *much less*, *less*, *normal*, *more*, *much more*. The fuzzy linguistic variable for distance to centroid, node degree, and distance to BS has the membership degree division as *low*, *medium*, *high*. Moreover, the linguistic variables for the fuzzy output variable *chance* are *very low*, *low*, *rather low*, *medium*, *high*, *rather high*, *very high*. Then, the membership functions of the inputs and output can be formulated by referring [17,34] and experimental experiences, which are shown in Figure 3 and Figure 4, respectively.

After fuzzification, the fuzzy inference engine is used to calculate the value of the output variable based on the *IF–THEN* rules. Instead of constructing the rule base according to the experts’ knowledge and empirical data, DPFCP generates rules using particle swarm optimization based on four inputs: residual energy, distance to centroid, node degree, and distance to BS. Because membership values for the inputs are 5, 3, 3 and 3, respectively, there are 5 × $3^{3}\;$= 135 rules in the fuzzy rule base, which are shown in Table 2.

PSO is one of the best optimization algorithms whose inspiration comes from birds searching for food [32]. Compared with the other metaheuristic approaches, PSO has shown greater excellence in exploration and exploitation applications [13]. Moreover, PSO has also been widely used to solve the optimization problems for clustering and membership function adjustment, with many advantages such as easy implementation, availability to escape from local optima, and quick convergence [13,35,36,37]. In PSO, a candidate solution is abstracted as a particle, and a particle updates its position and velocity according to its local best $p{Best}_{i}$, and the global best gBest of all the particles, so as to achieve the optimum objectives. Moreover, a fitness function is defined to determine the values of each particle considering different parameters related to the objectives. So, each particle in PSO denoted by *Pi = (x_i_*_1_, *x_i_*_2_, *x_i_*_3_, …, *x_in_)* can be encoded as a string whose dimension is 135 according to the fuzzy output indicated by the seven membership functions *very low*, *low*, *rather low*, *medium*, *high*, *rather high*, *very high*. An illustration is given in Table 3.

Then, the rule base can be derived as:

Rule 1: if $E_{res}$ = much less, $E_{to}C$ = low, $N_{dn}$ = low, $D_{to}BS$ = low, then *chance* = rather low

Rule 2: if $E_{res}$ = much less, $E_{to}C$ = low, $N_{dn}$ = low, $D_{to}BS$ = medium, then *chance* = low

Rule 3: if $E_{res}$ = much less, $E_{to}C$ = low, $N_{dn}$ = low, $D_{to}BS$ = high, then *chance* = very low

Rule 4: if $E_{res}$ = much less, $E_{to}C$ = low $N_{dn}$ = medium, $D_{to}BS$ = low, then *chance* = low

Rule 5: if $E_{res}$ = much less, $E_{to}C$ = low, $N_{dn}$ = medium, $D_{to}BS$ = medium, then *chance* = low

Rule 6: if $E_{res}$ = much less, $E_{to}C$ = low, $N_{dn}$ = medium, $D_{to}BS$ = high, then *chance* = very low

⋮

Rule 135: if $E_{res}$ = very high, $E_{to}C$ = high, $N_{dn}$ = high, $D_{to}BS$ = high, then *chance* = high.

In order to obtain the optimal rules for selection of the best CHs, the fitness function is defined to find the global solution that maximizes the *chance* value by the defuzzifier, which can be expressed by:

(5) $$\mathrm{Maximize}:\;\mathrm{fitness}=\frac{\sum_{i=1}^{k}c_{i}\times \mu_{i}}{\sum_{i=1}^{k}\mu_{i}}$$

where $c_{i}$ indicates the output of rule *i*, *k* is the number of rules, i.e., 135, and $\mu_{i}$ denotes the centroid of the output membership function. Let the popular size be $N_{p};$ then, the quality of particles is evaluated one by one using the fitness function so as to obtain the individual best ${pBest}_{i}(1\leq i\leq N_{p})$ and global the best gBest*,* which mean the best values of individuals and the population. Subsequently, the position and velocity of the particles are updated according to Equations (6) and (7), respectively.

(6) $$X_{}^{t+1}=X_{}^{t}+V_{}^{t+1}$$

(7) $$V_{}^{t+1}=\omega \times V_{}^{t}+c_{1}r_{1}(pBest_{}-X_{}^{t})+c_{2}r_{2}(gBest-X_{}^{t})$$

where *t* is the number of the iteration and ω indicates the inertial weight within (0, 1), *c*_1_, *c*_2_ denote acceleration coefficients, usually set to 2.0 [38], while *r*_1_, *r*_2_ are random numbers within the interval [0, 1]. Moreover, the inertial weight is adaptively adjusted for the optimal local and global search capability of the particles, which can be expressed by:

(8) $$\omega =\omega_{\max}-\frac{\omega_{\max}-\omega_{mi}{}_{n}}{t_{\max}}\times t$$

where $t_{\max}$ indicates the predetermined number of the iteration, $\omega_{\max},\;\omega_{\min}$ denote the maximum and minimum values of ω, which are usually set to 0.9 and 0.4, respectively [39].

**Figure 5:**
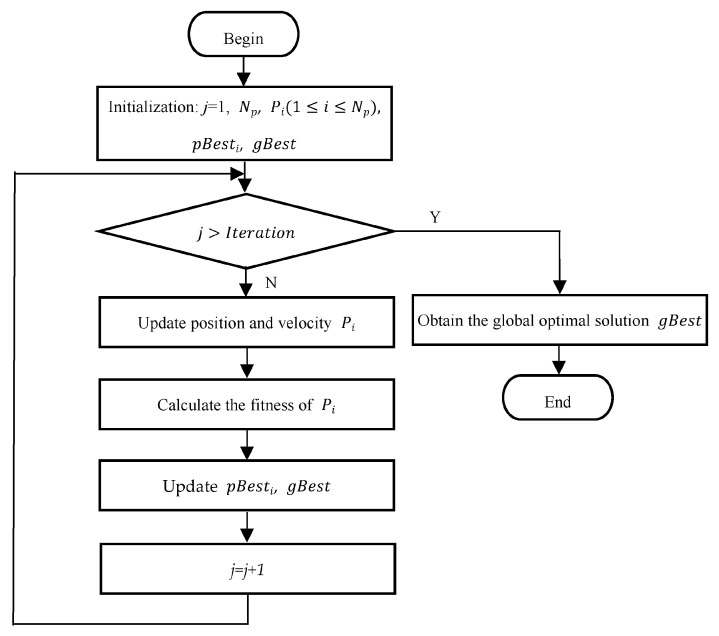
Flowchart of PSO used for rule generation.

Through iterative updating, the global optimal solution gBest can be finally achieved. The flowchart of PSO used to generate rules in DPFCP is presented in Figure 5.

As shown in Figure 5, the size of the population, $N_{p}$, and the number of iterations *Iteration*, are pre-defined, and the particles are initialized using the uniform random numbers with their respective range values, as shown in Table 2. At the same time, the individual best ${pBest}_{i}$ and global best gBest are set to zero. Afterwards, the population is updated by using Equations (6) and (7), and the quality of the particles is evaluated using the fitness function in Equation (5). Accordingly, the values of ${pBest}_{i}$ and gBest are also updated based on the fitness values of particles. Iteratively, the particles converge to the global optima. Thus, the global best gBest is obtained, and can be decoded to achieve the optimal fuzzy rules. More importantly, the optimization process only needs to be performed one time for a specific application.

Finally, the output crisp chance of the defuzzifier is derived, which comes from the last fitness value of gBest. After obtaining the crisp output, each node broadcasts a message including this output value to compete with its neighbors to be a CH, and also saves the values from its neighbors. Finally, the nodes with higher output values become CHs and the other nodes turn into CMs.

### 4.2. Cluster Formation

In the proposed protocol, each CM selects an appropriate CH to form energy efficient and balanced clusters according to a novel cost function considering not only distance to CH, but also residual energy and deviation of the CH’s number of members, which can be expressed as:

(9) $${cost}_{i}=\frac{Eres(i)}{D_{to}CH(j)\times |ND({CH}_{j})-\frac{\sum_{j=1}^{k}ND({CH}_{j})}{k}\;|}$$

where $D_{to}CH(j)$ indicates the distance between CM *i* and its candidate CH *j*, and *k* is the number of its candidate CHs. Moreover, $ND({CH}_{j})$ indicates the node degree of CM_i_’s candidate CH *j*. Firstly, each final selected CH broadcasts a message including its ID and node degree within its communication range. Each CM calculates its cost value according to Equation (8) to determine which cluster to join. If *k* = 0, i.e., no message is received from any CH, the CM will announce itself as a CH. Accordingly, optimal clusters are formed with CHs and their corresponding CMs possessing higher cost values. Data transmission after cluster formation is also considered to be a crucial task for energy reduction in the WSN because improper data transmission results in data transmission failure or collision. To alleviate these problems, time division multiple access (TDMA) is used to transmit data from CMs to CHs.

### 4.3. On Demand Re-Clustering

In contrast to conventional round-by-round [4], energy triggered by the BS [25], or a specific algorithm to determine the round [10], in DPFCP, a distributed light mechanism is utilized to deal with the energy-consuming maintenance process based only on local information. Firstly, each CH remains the CH for a certain number of upcoming rounds. Once its residual energy reaches the average energy of the cluster, the CH announces the node with the second-largest *chance* as CH. Next is the node with the third-largest *chance*, etc. Only a small control packet is needed to perform the CH shift every time based on the saved *chance* values, which requires little computation and message overhead compared with other methods. Secondly, when any CH acts as a CH only in a round, it broadcasts a small control packet to its neighbor CHs instead of its CMs, and the neighbor CHs also forward the packet until all CHs receive the message to perform CH selection, cluster formation, and on-demand re-clustering again.

### 4.4. Time Complexity Analysis

In DPFCP, the time complexity consists of the time complexity of the CH selection, cluster formation, and on-demand re-clustering. DPFCP uses fuzzy logic to select CHs whose time complexity is $O(N\times N_{rule}),$ in which *N* is the number of nodes, and $N_{rule}$ indicates the number of rules. Four inputs are used in DPFCP and their membership values for the inputs are 5, 3, 3, and 3, so there are 135 rules in total. Moreover, the rules are optimized by PSO whose time complexity is $O((N_{i}+N_{rule})\times N_{p}),$ where $N_{i}$ is the dimension of particles, equal to 135, and $N_{p}$ indicates the population size. Thus, the total time complexity of CH selection equals $O((N_{i}+N_{rule})\times N_{p}+N\times N_{rule})$. For cluster formation, message exchange consists of CHs broadcasting their identity and non-CHs joining the CH, and TDMA advertisement, whose time complexity values are O(1), O(N−1), and O(N), respectively. As for on-demand re-clustering, one and *k_c_*-1 messages are needed to be processed for the CH shift in the cluster and re-clustering in the network, respectively, in the worst case, where *k_c_* is the number of clusters. Hence, the time complexity for on-demand re-clustering is $O(1+k_{c})$. Generally, $N_{i},N_{rule},\;k_{c}$ is much less than *N*; therefore, the time complexity of DPFCP is O(N).

## 5. Simulation Results

As presented in this section, simulation experiments were conducted in MATLAB 2020a to test the performance of DPFCP compared to that of LEACH [4], LEACH-SF [14], FLS-PSO [25], and KM-PSO [10] in terms of network lifetime, network throughput, standard deviation of residual energy, and energy consumption. Two scenarios are considered for the proposed protocol and its counterparts.

In Scenario #1, 100 sensor nodes are randomly scattered in a target area of 100 m × 100 m, and the BS is located in the center of the area, i.e., at (50, 50). In Scenario #2, 300 sensor nodes randomly distributed in the 500 m × 500 m network, and the position of the BS is also taken as the center of the area, i.e., at (250, 250). The initial energy of the nodes is the same, namely 1 J in both of the scenarios. Moreover, the number of clusters is set to 5% or 10% of the total number of nodes in both of the scenarios, which is similar to most of the clustering protocols [25,37]. The other parameters for simulations are summarized in Table 4.

In order to reduce the test error and provide a reliable confidence interval in the simulation process, each scenario is run 50 times, and the average of these instances of data is used for plotting the results of the protocols. The detailed test results are shown and analyzed as follows.

### 5.1. Network Lifetime

**Figure 6:**
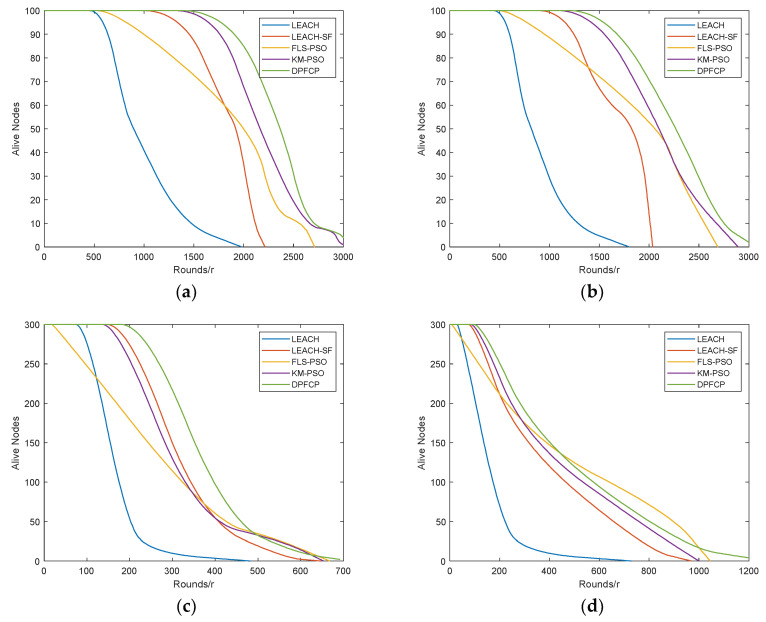
Comparison of the network lifetime: (**a**) Scenario #1, 10% CH, (**b**) Scenario #1, 5% CH, (**c**) Scenario #2, 10% CH, (**d**) Scenario #2, 5% CH.

Usually, the number of surviving nodes represents the lifespan of the network, and three metrics are used for performance measurement, i.e., FND (first node dies), HND (half nodes die), and LND (last node dies). The test results in different scenarios are shown in Table 5 and Figure 6.

As seen in Figure 6, the performance of LEACH, LEACH-SF, FLS-PSO, KM-PSO, and DPFCP in Scenario #1 is superior to that in Scenario #2, which confirms the poor scalability of the clustering protocol due to its single-hop communication mode. Of course, this is why routing protocols are another significant challenge for WSNs. Even so, DPFCP performs the best in both the small-scale network and large-scale network. According to Table 5, the FND of LEACH occurs at 649, 584, 98, and 153 rounds in different scenarios, and that of LEACH-SF is 1382, 1173, 197, and 98 rounds. For FLS-PSO, it occurs at 754, 682, 24, and 10 rounds, while it is 1728, 1469, 173, and 110 rounds for KM-PSO. The FND of DPFCP occurs at 1865, 1594, 239, and 126 rounds, respectively. LEACH randomly selects the CHs, and some nodes with less residual energy may become CHs, which results in premature death of a certain node. Although FLS-PSO utilizes the fuzzy logic system to make decisions about the final CHs, its random selection of candidate CHs, like in LEACH, may also make a node with low residual energy be a CH. Furthermore, the node becomes a CH in multiple rounds due to the on-demand re-clustering mechanism. Thus, FLS-PSO is the earliest to have a dead node. Although the FND of LEACH-SF is later than that of FLS-PSO, its node death rate is much faster than that of FLS-PSO, KM-PSO, and DPFCP, because the intra-cluster energy balance is not considered in LEACH-SF. On the contrary, FLS-PSO and KM-PSO achieve a better lifetime than LEACH-SF by taking a second CH to share data collection and the formation of uniform clusters based on node degree, respectively. Overall, DPFCP considers the residual energy, node degree, distance to BS, and distance to centroid, and utilizes the fuzzy logic system to balance these parameters with its distributed characteristic; thus, it has the longest lifetime. In summary, in Scenario #1 and Scenario #2, the performance of DPFCP in extending the network lifetime is 51.3%, 54.03%, 40.91%, and 38.54% higher than that of LEACH; 23.72%, 25.29%, 11.73%, and 22.03% higher than that of LEACH-SF; 23.54%, 22.61%, 19.13%, and 16.49% higher than that of FLS-PSO; and 7.36%, 6.55%, 14.65%, and 15.43% higher than that of KM-PSO.

### 5.2. Energy Consumption

**Figure 7:**
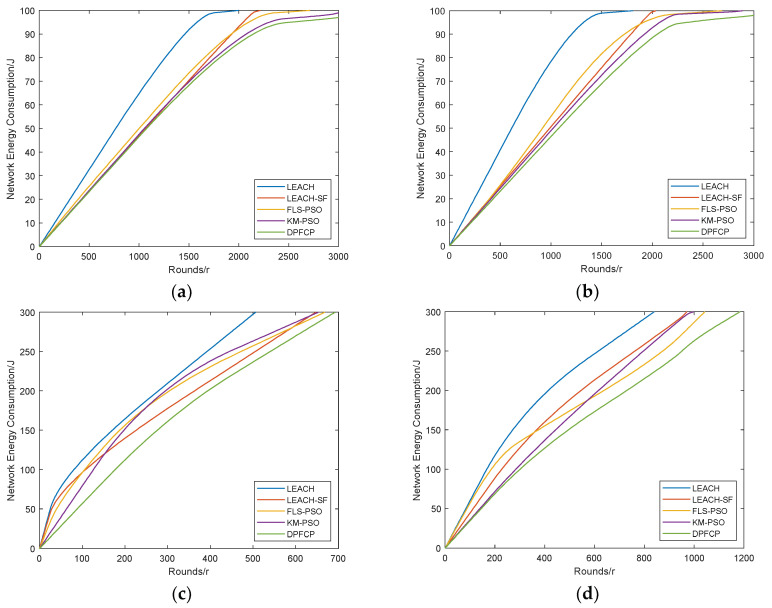
Comparison of the network energy consumption: (**a**) Scenario #1, 10% CH, (**b**) Scenario #1, 5% CH, (**c**) Scenario #2, 10% CH, (**d**) Scenario #2, 5% CH.

The energy of sensor nodes is consumed for data receiving, aggregating, and transmitting, while the energy consumption for calculating and sleeping is ignored. Then, the total energy consumption of the network is tested, and the comparison results of DPFCP with LEACH, LEACH-SF, FLS-PSO, and KM-PSO in the two scenarios are shown in Figure 7.

It can be observed from Figure 7 that DPFCP outperforms LEACH, LEACH-SF, FLS-PSO, and KM-PSO with respect to total energy consumption. In other words, the proposed protocol reduces the network energy consumption not only in the small network, but also in the large network because of its fully distributed characteristic. As the sensor nodes are nominated to their nearest CH and the selected CHs are close as possible to the BS, all the CHs and CMs consume less energy; as a result, the network energy consumption in DPFCP becomes lower than that in the others. This is due to the fact that DPFCP simultaneously considers energy consumption of CMs and CHs by reducing the distance among CMs, CHs, and the BS. However, long-distance transmission exists between CMs and CHs, as well as between CHs and the BS, resulting in the worst energy consumption performance. For LEACH-SF and KM-PSO, distance-based clusters are formed by fuzzy c-means and k-means, respectively. Moreover, CH selection is achieved by the fuzzy logic system with the same descriptors. However, PSO-based centroid decisions in KM-PSO can reduce greater intra-cluster energy consumption compared to ABC-based CH selection in LEACH-SF. In addition, FLS-PSO consumes more energy than LEACH-SF and KM-PSO because of its random selection of CH candidates in the early stage of network operation. Due to its on-demand re-clustering mechanism, the message overhead used for cluster formation is reduced, and the total energy consumption is then less than that of LEACH-SF and KM-PSO. When half of the network energy is consumed in both scenarios, the running rounds of DPFCP are 29.34%, 42.23%, 37.13%, and 44.12% higher than those of LEACH; 2.38%, 13.64%, 21.04%, and 26.34% higher than those of LEACH-SF; 8.29%, 18.85%, 34.29%, and 23.2% higher than those of FLS-PSO; and 3.49%, 6.79%, 30.51%, and 11.47% higher than those of KM-PSO.

### 5.3. Network Throughput

**Figure 8:**
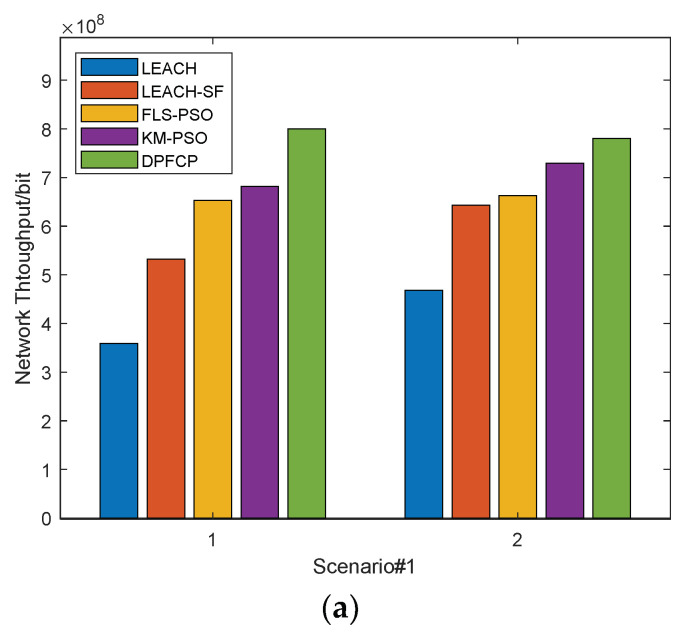

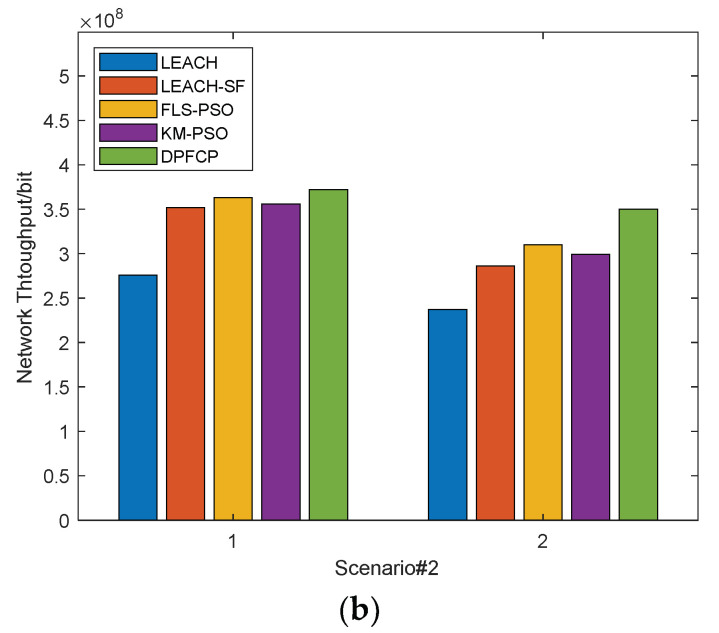
Comparison of the network throughput: (**a**) Scenarios #1 and #2, 10% CH, (**b**) Scenarios #1 and #2, 5% CH.

The network throughput equals the number of data received by the BS throughout the entire network lifecycle. The higher the network throughput, the more data packets the BS receives, which indicates the better the network performance. Obviously, as the network runs, the network throughput is increasing. The test results of the network throughput are depicted in Figure 8.

From Figure 8, it can be seen that in both scenarios, DPFCP outperforms LEACH, LEACH-SF, FLS-PSO, and KM-PSO in terms of network throughput. The reason is that the number of data received by the BS depends on both the energy consumption and the network lifetime. In Scenario #1 and Scenario #2, the network throughput of DPFCP is improved by 55.63%, 40.03%, 25.81%, and 32.29% compared to that of LEACH; by 30.64%, 16.72%, 5.49%, and 19.34% compared to that of LEACH-SF; by 19.73%, 16.35%, 3.65%, and 12.78% compared to that of FLS-PSO; and by 15.21%, 5.94%, 4.47%, and 15.18% compared to that of KM-PSO. In LEACH, random CH selection and long-distance communication result in high energy consumption and a short lifespan, so it has the smallest network throughput. LEACH-SF can generate optimal clusters using fuzzy c-means and the fuzzy logic system with ABC optimization, which prolongs the network and also improves the network throughput. Similarly, FLS-PSO forms appropriate clusters by utilizing the fuzzy logic system with membership optimization by PSO, while the on-demand re-clustering mechanism is employed to further reduce energy consumption. Thus, the overall performance of network throughput is better than that of LEACH-SF. For KM-PSO, more data are transmitted to the BS because it forms energy-efficient clusters based on k-means and PSO, and the primary CH and secondary CH are selected to reduce intra-cluster energy consumption.

### 5.4. Standard Deviation of Residual Energy

**Figure 9:**
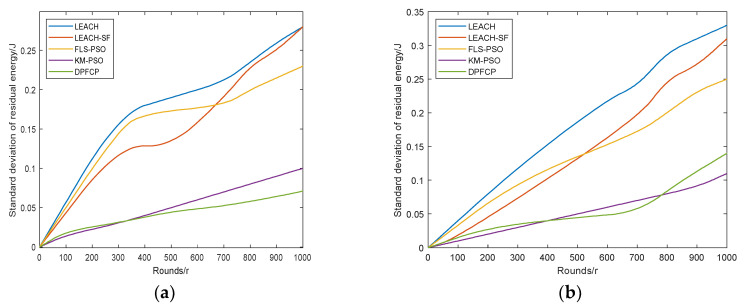

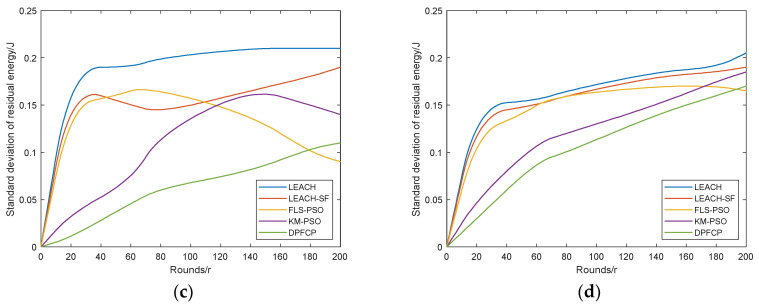
Comparison of standard deviation of residual energy: (**a**) Scenario #1, 10%CH, (**b**) Scenario #1, 5%CH, (**c**) Scenario #2, 10%CH, (**d**) Scenario #2, 5%CH.

To verify the balance of energy consumption in the network, the standard deviation of residual energy is tested in the two scenarios. The results are shown in Figure 9.

As shown in Figure 9, the standard deviation of the network residual energy in DPFCP is usually lower than that of the other four protocols. Moreover, its change is smaller than that of other protocols, indicating its better performance in balanced energy consumption. In Scenarios #1 and #2, the average standard deviation of the network residual energy in DPFCP is 79.83%, 54.41%, 70.54%, and 42.75% lower than that of LEACH; 70.54%, 49.25%, 60.07%, and 41.6% lower than that of LEACH-SF; 74.39%, 47.18%, 53.66, and 40.87% lower than that of FLS-PSO; and 10.41%, 3.82%, 43.81%, and 19.53% lower than that of KM-PSO. Residual energy is not considered for CH selection in LEACH and FLS-PSO, resulting in an energy consumption imbalance for CHs. Moreover, non-CH nodes join the nearest CH in LEACH based only on the distance, which may form uneven clusters whose energy consumption is also unequal. By comparison, in FLS-PSO, uniform clusters are formed by the fuzzy logic system with the descriptors of node degree, node centroid, and packet drop probability. Although LEACH-SF considers residual energy to select CHs, increasing the distance between CHs and BS results in a sharp growth in CH load. Accordingly, imbalanced energy consumption occurs in the network. KM-PSO not only considers the energy of nodes, but also adds the SCH during clustering, which is specifically used to transmit data to the BS, so its energy consumption is more balanced than that of LEACH-SF and FLS-PSO. However, in a large-scale network, the distance between the SCH and PCH is larger than that in a small-scale network, resulting in imbalanced intra-cluster energy consumption. By comparison, the on-demand re-clustering mechanism based on an energy threshold in FLS-PSO can still balance the CHs’ energy consumption, so its performance in Scenario #2 is better than that of KM-PSO. Moreover, in DPFCP, residual energy and the CH’s node degree are considered to make non-CHs join an appropriate CH for balanced intra-cluster energy consumption. Furthermore, the on-demand re-clustering mechanism based on local information employed in DPFCP balances the CHs’ energy consumption compared to KM-PSO. Therefore, the overall energy consumption of DPFCP is more balanced than that of PLL-PSO.

## 6. Conclusions

In this paper, an effective DPFCP protocol to form balanced clusters and maximize the network lifetime is proposed. In DPFCP, an optimized fuzzy logic system is applied to select proper clusters based on the local information of sensor nodes including residual energy, node degree, distance to the BS, and distance to the centroid. In order to achieve this objective, a PSO algorithm is utilized to optimize the fuzzy rules. Moreover, the optimization procedure should be performed only once before DPFCP operates for a specific application. In addition, non-CHs join the appropriate CHs to form energy-balanced clusters based on the parameters such as distance to CH, residual energy, and deviation in the CH’s number of members. Furthermore, an on-demand re-clustering mechanism is utilized to maintain local clusters and the network for energy saving. According to the simulation results, DPFCP can efficiently balance the network energy consumption and maximize the network lifetime. It outperforms LEACH, LEACH-SF, FLS-PSO, and KM-PSO in terms of energy consumption, throughput, standard deviation of residual energy, and network lifetime. Specifically, compared with LEACH, LEACH-SF, FLS-PSO, and KM-PSO, the network lifetime of DPFCP is increased by 52.66%, 24.5%, 23.07%, and 6.95%, and 39.72%, 16.88%, 17.81%, and 15.04%, in Scenarios #1 and #2, respectively.

Although this work achieves good results with respect to network lifetime, throughput, energy efficiency, and energy balance, there are still some limitations to be addressed in the future. In DPFCP, CHs communicate with the BS in single-hop mode to limit the network scalability, and the multi-hop routing method can be considered to extend it for a larger-scale network. In addition, the proposed protocol has been designed for proactive networks; thus, it can be extended to support a reactive network to take different failures and intrusions of the network into account. Finally, the tests are performed based on the ideal network model; thus, practical scenarios will be used to test the proposed protocol for applicability verification.

## Figures and Tables

**Table 1:** Summary of existing protocols.

Protocol	Method	Network Type	FLS						Objectives
			Model	Input Parameters	Rules	Optimization	Output	Maintaneance	
[28]	Distributed	Homogenous	Mamdani	Distance from BS Node density Battery level	Fixed	—	Weight value	Periodic	Reduce network power consumption and increase network lifetime
[29]	Distributed	Homogenous	Mamdani	Residual energy Node density Distance to BS	Fixed	—	Competitive radius Rank	Periodic	Prolong the lifetime of the network and solve the hot spot problem
[30]	Centralized	Homogenous and Heterogeneous	Mamdani	Node energy Node concentration Node centrality Energy Distance to BS Distance to CH	Fixed	—	Chance	Periodic	Maximizing network lifetime
[31]	Centralized and Distributed	Homogenous	Mamdani	Residual energy Distance to BS Node density Cluster status	Fixed	—	Chance	Periodic	Enhance the lifetime of the sensor nodes
[10]	Centralized	Homogenous	Mamdani	FLS1-PCH Residual energy Distance to cluster center Distance to BS FLS2-SCH Residual energy Distance to PCH	Fixed	—	Chance	Periodic	Improve the network energy efficiency and boost the network throughput
[32]	Centralized and Distributed	Homogenous	Mamdani	Residual energy Distance between the node and cluster centre	Fixed	—	Fitness	Periodic	Enhance network lifetime
[14]	Centralized	Homogenous	Sugeno	Residual energy Distance between the node and cluster centre	Tuned	ABC for rules	Impact Factor (IF)	Periodic	Prolong network lifetime
[25]	Centralized and Distributed	Homogenous	Mamdani	Node degree Node centrality Packet drop probability	Fixed	PSO for membership function	Chance	On demand	Improve the network energy efficiency and throughput
The proposed	Distributed	Homogenous	Mamdani	Residual energy Node degree Distance to BS Distance to the centroid	Tuned	PSO for rules	Chance	On demand	Maximizing network lifetime and balance energy consumption

**Table 2:** IF–THEN rules.

No.	$E_{res}$	$E_{to}C$	$N_{dn}$	$D_{to}BS$	*Chance*
**1**	much less	low	low	low	$c_{1}$
**2**	much less	low	low	medium	$c_{2}$
**3**	much less	low	low	high	$c_{3}$
**4**	much less	low	medium	low	$c_{4}$
**5**	much less	low	medium	medium	$c_{5}$
**6**	much less	low	medium	high	$c_{6}$
**⋮**	**⋮**	**⋮**	**⋮**	**⋮**	**⋮**
**135**	much more	high	high	high	$c_{135}$

**Table 3:** Illustration of particle presentation in DPFCP.

*P_i_*	*3*	*2*	*1*	*2*	*2*	*1*	*⋯*	*5*
	$x_{i1}$ $c_{1}$	$x_{i2}$ $c_{2}$	$x_{i3}$ $c_{3}$	$x_{i4}$ $c_{4}$	$x_{i5}$ $c_{5}$	$x_{i6}$ $c_{6}$	⋯	$x_{i135}$ $c_{135}$
**1**	very low	very low	very low	very low	very low	very low	⋯	very low
**2**	low	low	low	low	low	low	⋯	low
**3**	rather low	rather low	rather low	rather low	rather low	rather low	⋯	rather low
**4**	medium	medium	medium	medium	medium	medium	⋯	medium
**5**	high	high	high	high	high	high	⋯	high
**6**	rather high	rather high	rather high	rather high	rather high	rather high	⋯	rather high
**7**	very high	very high	very high	very high	very high	very high	⋯	very high

**Table 4:** Network parameters.

Parameters	Scenario #1	Scenario #2
Number of nodes	100	300
Initial energy	1 J	1 J
*E_elec_*	50 (nJ/bit)	50 (nJ/bit)
$E_{0}$	5(nJ/bit)	5(nJ/bit)
$\varepsilon_{\mathrm{fs}}$	10 (pJ/bit/m^2^)	10 (pJ/bit/m^2^)
$\varepsilon_{\mathrm{mp}}$	0.0013 (pJ/bit/m^4^)	0.0013 (pJ/bit/m^4^)
$d_{0}$	87.7 m	87.7 m
$E_{pDb}$	5 nJ/bit	5 nJ/bit
Data packet size	4000 bits	4000 bits
Control packet size	200 bits	200 bits
Network area	100 m × 100 m	500 m × 500 m
BS Location	x = 50, y = 50	x = 250, y = 250
$N_{p}$	30	30
Iteration	100	100

**Table 5:** Comparison of FND, HND and LND.

	Protocols		LEACH	LEACH-SF	FLS-PSO	KM-PSO	DPFCP
Items							
Scenario #1	100 nodes CH:10%	FND	649	1382	754	1728	1865
		HND	854	1942	2094	2131	2342
		LND	1967	2214	2712	2954	3109
	100 nodes CH:5%	FND	584	1173	682	1469	1594
		HND	817	1904	2120	2090	2305
		LND	1792	2036	2689	2893	3016
Scenario #2	300 nodes CH:10%	FND	98	197	24	173	239
		HND	209	365	387	352	426
		LND	493	647	668	653	692
	300 nodes CH:5%	FND	36	98	10	110	126
		HND	153	264	327	303	350
		LND	829	970	1042	997	1193

## Data Availability

Not applicable.
